# Seven-Year Neurodevelopmental Scores and Prenatal Exposure to Chlorpyrifos, a Common Agricultural Pesticide

**DOI:** 10.1289/ehp.1003160

**Published:** 2011-04-21

**Authors:** Virginia Rauh, Srikesh Arunajadai, Megan Horton, Frederica Perera, Lori Hoepner, Dana B. Barr, Robin Whyatt

**Affiliations:** 1 Heilbrunn Center for Population and Family Health, Mailman School of Public Health,; 2 Department of Biostatistics, Mailman School of Public Health,; 3 Sergievsky Center, and; 4 Columbia Center for Children’s Environmental Health, Mailman School of Public Health, Columbia University, New York, New York, USA; 5 Emory University, Atlanta, Georgia, USA

**Keywords:** chlorpyrifos, neurodevelopment, pesticides

## Abstract

Background: In a longitudinal birth cohort study of inner-city mothers and children (Columbia Center for Children’s Environmental Health), we have previously reported that prenatal exposure to chlorpyrifos (CPF) was associated with neurodevelopmental problems at 3 years of age.

Objective: The goal of the study was to estimate the relationship between prenatal CPF exposure and neurodevelopment among cohort children at 7 years of age.

Methods: In a sample of 265 children, participants in a prospective study of air pollution, we measured prenatal CPF exposure using umbilical cord blood plasma (picograms/gram plasma) and 7-year neurodevelopment using the Wechsler Intelligence Scale for Children, 4th edition (WISC-IV). Linear regression models were used to estimate associations, with covariate selection based on two alternate approaches.

Results: On average, for each standard deviation increase in CPF exposure (4.61 pg/g), Full-Scale intelligence quotient (IQ) declined by 1.4% and Working Memory declined by 2.8%. Final covariates included maternal educational level, maternal IQ, and quality of the home environment. We found no significant interactions between CPF and any covariates, including the other chemical exposures measured during the prenatal period (environmental tobacco smoke and polycyclic aromatic hydrocarbons).

Conclusions: We report evidence of deficits in Working Memory Index and Full-Scale IQ as a function of prenatal CPF exposure at 7 years of age. These findings are important in light of continued widespread use of CPF in agricultural settings and possible longer-term educational implications of early cognitive deficits.

Each year, thousands of new chemicals are released in the United States, with very little documentation about potential long-term human health risks (Landrigan et al. 2002). First registered in 1965 for agricultural and pest control purposes, chlorpyrifos (CPF; 0,0-diethyl-0-3,5,6-trichloro-2-pyridyl phosphorothioate) is a broad-spectrum, chlorinated organophosphate (OP) insecticide. Before regulatory action by the U.S. Environmental Protection Agency (EPA) to phase out residential use beginning in 2000, CPF applications were particularly heavy in urban areas, where the exposed populations included pregnant women (Berkowitz et al. 2003; Whyatt et al. 2002, 2003). In a sample of pregnant women in New York City (Perera et al. 2002) detectable levels of CPF were found in 99.7% of personal air samples, 100% of indoor air samples, and 64–70% of blood samples collected from umbilical cord plasma at delivery (Whyatt et al. 2002).

Early concerns about the possible neurotoxicity of OP insecticides for humans derived from rodent studies showing that prenatal and early postnatal exposures to CPF were associated with neurodevelopmental deficits, and these effects have been seen at exposure levels well below the threshold for systemic toxicity caused by cholinesterase inhibition in the brain (e.g., Slotkin and Seidler 2005). Evidence has accumulated over the past decade showing that noncholinergic mechanisms may play a role in the neurotoxic effects of CPF exposure in rodents, involving disruption of neural cell development, neurotransmitter systems (Aldridge et al. 2005; Slotkin 2004), and synaptic formation in different brain regions (Qiao et al. 2003). Such developmental disruptions have been associated with later functional impairments in learning, short-term working memory, and long-term reference memory (Levin et al. 2002).

In humans, OPs have been detected in amnionic fluid (Bradman et al. 2003) and are known to cross the placenta (Richardson 1995; Whyatt et al. 2005), posing a threat to the unborn child during a period of rapid brain development. Using urinary metabolites as the biomarker of exposure, several different birth cohort studies have reported that prenatal maternal nonspecific OP exposure was associated with abnormal neonatal reflexes (Engel et al. 2007; Young et al. 2005), mental deficits and pervasive development disorder at 2 years (Eskenazi et al. 2007), and attention problem behaviors and a composite attention-deficit/hyperactivity  disorder indicator at 5 years of age (Marks et al. 2010).

Using a different biomarker of exposure (the parent compound of CPF in umbilical cord plasma), we have previously reported (in the same cohort as the present study) significant associations between prenatal exposure to CPF (> 6.17 pg/g) and reduced birth weight and birth length (Whyatt et al. 2004), increased risk of small size for gestational age (Rauh V, Whyatt R, Perera F, unpublished data), increased risk of mental and motor delay (< 80 points) and 3.5- to 6-point adjusted mean decrements on the 3-year Bayley Scales of Infant Development (Rauh et al. 2006), and evidence of increased problems related to attention, attention deficit hyperactivity disorder, and pervasive developmental disorder as measured by the Child Behavior Checklist at 2–3 years (Rauh et al. 2006). Taken together, these prospective cohort studies show a consistent pattern of early cognitive and behavioral deficits related to prenatal OP exposure, across both agricultural and urban populations, using different biomarkers of prenatal exposure.

We undertook the present study to identify the developmental consequences of prenatal exposure to CPF in a sample of New York City children at 7 years of age. Given the mechanisms proposed in the rodent literature, and early findings from prospective human studies involving nonspecific OP exposures, we hypothesized that prenatal exposure to CPF would be associated with neurodevelopmental deficits persisting into the early school years, when more refined neuropsychological tests are available to identify particular functional impairments.

## Materials and Methods

*Participants and recruitment.* The subjects for this report are participants in an ongoing prospective cohort study (Columbia Center for Children’s Environmental Health) of inner-city mothers and their newborn infants (Perera et al. 2002). The cohort study was initiated in 1997 to evaluate the effects of prenatal exposures to ambient pollutants on birth outcomes and neurocognitive development in a cohort of mothers and newborns from low-income communities in New York City. Nonsmoking women (classified by self-report and validated by blood cotinine levels < 15 ng/mL), 18–35 years of age, who self-identified as African American or Dominican and who registered at New York Presbyterian Medical Center or Harlem Hospital prenatal clinics by the 20th week of pregnancy, were approached for consent. Eligible women were free of diabetes, hypertension, known HIV, and documented drug abuse and had resided in the area for at least 1 year. The study was approved by the Institutional Review Board of Columbia University. Informed consent was obtained from all participating mothers, and informed assent was obtained from all children as well, starting at 7 years of age.

Of 725 consenting women, 535 were active participants in the ongoing cohort study at the time of this report, and 265 of their children had reached the age of 7 years with complete data on the following: *a*) prenatal maternal interview data, *b*) biomarkers of prenatal CPF exposure level from maternal and/or cord blood samples at delivery, *c*) postnatal covariates, and *d*) neurodevelopmental outcomes.

*Maternal interview and assessment.* A 45-min questionnaire was administered to each woman in her home by a trained bilingual interviewer during the third trimester of pregnancy and annually thereafter. From the interviews and medical records, the following sociodemographic and biomedical variables, among others, were available: race/ethnicity, infant sex, household income, maternal age, maternal completed years of education at child’s age 7 years, birth weight, gestational age, and self-reported maternal exposure to environmental tobacco smoke (ETS) during pregnancy.

We measured maternal nonverbal intelligence by the Test of Nonverbal Intelligence, 3rd edition (TONI-3) (Brown et al. 1997), a 15-min language-free measure of general intelligence, administered when the child was 3 years of age. The quality of the care-taking environment was measured by the Home Observation for Measurement of the Environment (HOME) inventory when the child was 3 years of age (Caldwell and Bradley 1979) to assess physical and interactive home characteristics. The mother report version of the Child Behavior Checklist for ages 6–18 years, a well-validated measure of child behavior problems occurring in the preceding 2 months (Achenbach and Rescorla 2001), was administered at 7 years as part of the larger cohort study.

*Biological samples and pesticide exposure.* A sample of umbilical cord blood (30–60 mL) was collected at delivery, and a sample of maternal blood (30–35 mL) was collected within 2 days postpartum by hospital staff. Portions were sent to the Centers for Disease Control and Prevention (Atlanta, GA) for analysis of CPF in plasma, as well as lead and cotinine, described in detail elsewhere (Perera et al. 2002; Whyatt et al. 2003). Methods for the laboratory assay for CPF, including quality control, reproducibility, and limits of detection (LODs), have also been previously published (Barr et al. 2002). In cases where the umbilical cord blood sample was not collected (12% of subjects), mothers’ values were substituted, using a formula previously derived from regression analyses (Whyatt et al. 2005). As previously reported, maternal and umbilical cord blood CPF concentrations were similar (arithmetic means ± SDs of 3.9 ± 4.8 pg/g for maternal blood and 3.7 ± 5.7 pg/g for cord blood) (Whyatt et al. 2005), and CPF levels in paired maternal and umbilical cord plasma samples were highly correlated (*r* = 0.76; *p* < 0.001, Spearman’s rank), indicating that CPF was readily transferred from mother to fetus during pregnancy. Prenatal blood lead levels were available for a subset of children (*n* = 89). ETS exposure, measured by maternal self-report, was validated by cotinine levels in umbilical cord blood, as described in detail elsewhere (Rauh et al. 2004). We measured polycyclic aromatic hydrocarbon (PAH) exposure by personal air monitoring during the third trimester, using a previously described method, and excluding poor-quality samples (Perera et al. 2003). As previously described (Perera et al. 2003), we computed a composite log-transformed PAH variable from the eight correlated PAH air concentration measures (*r*-values ranging from 0.34 to 0.94; all *p*-values < 0.001 by Spearman’s rank).

In the larger cohort study, > 40% of CPF exposure values for combined maternal and umbilical cord blood samples were below the LOD. Using a method suggested by Richardson and Ciampi (2003), we made a distributional assumption for the exposure variable (log-normal CPF), computed the expected value of the exposure (*E*) for all nondetects [*E*(*X*/*X* < LOD)], and assigned this value to all nondetects.

*Measures of neurodevelopment.* For the 7-year assessment, we selected the Wechsler Intelligence Scale for Children, 4th edition (WISC-IV), because of its revised structure based on the latest research in neurocognitive models of information processing (Wechsler 2003). The WISC-IV is sensitive to low-dose neurotoxic exposures, as demonstrated by studies of lead toxicity in 6- to 7.5-year-old children (Chiodo et al. 2004; Jusko et al. 2008; Rothenberg and Rothenberg 2005). The instrument measures four areas of mental functioning that are associated with, but distinct from, overall intelligence quotient (IQ) and is sensitive to cognitive deficits related to learning and working memory, which have been linked to CPF exposure in rodent studies (e.g., Levin et al. 2002). Each standardized scale has a mean of 100 and SD of 15. The Verbal Comprehension Index is a measure of verbal concept formation, a good predictor of school readiness (Hecht et al. 2000; Wechsler 2003); the Perceptual Reasoning Index measures nonverbal and fluid reasoning; the Working Memory Index assesses children’s ability to memorize new information, hold it in short-term memory, concentrate, and manipulate information; the Processing Speed Index assesses ability to focus attention and quickly scan, discriminate, and sequentially order visual information; and the Full-Scale IQ score combines the four composite indices. The General Ability Index score is a summary score of general intelligence, similar to Full-Scale IQ, but excludes contributions from both Working Memory Index and Processing Speed Index (Wechsler 2003). WISC-IV scores may be influenced by socioeconomic background and/or child behavior problems particularly those related to anxiety (Wechsler 2003).

*Data analysis.* We conducted all analyses using the statistical program R (R Development Core Team 2010). We treated CPF exposure level (picograms per gram) as a continuous variable. We natural log (ln) transformed the WISC-IV Composite Index scores to stabilize the variance and to improve the linear model fit, based on regression diagnostics. Unadjusted correlation analyses were used to explore associations between CPF exposure and WISC-IV scores. We constructed smoothed cubic splines to explore the shape of the functional relationships between CPF exposure and each of the log-transformed WISC-IV indices. We compared the models in which CPF is entered as a single continuous outcome with those in which CPF is modeled using B-splines, using the Davidson–MacKinnon *J*-test for comparing nonnested models (Davidson and MacKinnon 1981).

Demographic, biomedical, and chemical exposure variables collected for the larger cohort study were available for possible inclusion in the present analysis. We used two different approaches for covariate selection and model fitting, for the purpose of determining the robustness of our results with respect to alternate methods. Covariates were initially selected based on prior literature and retained in the models if associated with either CPF exposure or the WISC-IV scales (*p* < 0.10 in univariate analyses). Multiple linear regression was used to test the effects of prenatal CPF exposure on each 7-year WISC-IV Index. We examined residuals for normality and homoscedasticity and detected no problems. In addition, we employed the least absolute shrinkage and selection operator (LASSO), a shrinkage with selection procedure that provides a more parsimonious approach to covariate selection and model fitting (Houwelingen 2001; Tibshirani 1996). This method minimizes the usual sum of squared errors, with a bound on the sum of the absolute values of the coefficients, thereby shrinking very unstable estimates toward zero, excluding redundant/irrelevant covariates, and avoiding overfitting (Zhao and Yu 2006). We used Sobel’s indirect test to assess the influence of child behaviors on the estimates of CPF effect (MacKinnon et al. 2002; Sobel 1982). We used Sobel’s indirect test to assess mediation (MacKinnon et al. 2002; Sobel 1982). Interaction terms including CPF and each additional covariate were tested in the models. Effect estimates, 95% confidence intervals (CIs), and *p*-values were calculated for all analytic procedures. Results were considered significant at *p* < 0.05.

## Results

The retention rate for the full cohort was 82% at the 7-year follow-up, with no significant sociodemographic differences between subjects retained in the study and those lost to follow-up (data not shown). Table 1 lists characteristics of the study sample with complete data on all variables (*n* = 265). Study families were predominantly low income, with 31% of mothers failing to complete high school by child’s age 7 years, and 66% never married. The sample was largely full term (only 4% of children in the sample were < 37 weeks gestational age at delivery) and included very few low-birth-weight infants because *a*) we excluded high-risk pregnancies from the study cohort, and *b*) the timing of air monitoring in the third trimester of pregnancy eliminated early deliveries.

**Table 1:** Demographic characteristics of the sample at 7-year follow-up (*n* = 265).

Characteristic	*n* (%) or mean ± SD (range)
Home quality*a*	40.23 ± 4.81 (23–52)
Income	
< $20,000	138 (52)
≥ $20,000	127 (48)
Maternal education*b*	
Years	12.22 ± 2.58 (1–20)
< High school degree	82 (31)
High school degree	183 (69)
Maternal IQ*c*	85.97 ± 13.46 (60–135)
Maternal race/ethnicity*d*	
Dominican	146 (55)
African American	119 (45)
Marital status	
Never married	175 (66)
Ever married	90 (34)
Child sex	
Male	117 (44)
Female	148 (56)
Gestational age (weeks)	39.3 ± 1.5 (30–43)
Birth weight (g)	3389.8 ± 493.5 (1,295–5,110)
Child age at testing (months)	85.97 ± 2.65 (74.90–101.5)
Prenatal chemical exposures	
ETS*e*	
Exposed	93 (35)
Not exposed	172 (65)
Cotinine (ng/mL)*f*	0.25 ± 0.92 (0.01–8.78)
Lead (μg/dL)*f*	1.09 ±.88 (0.15–7.45)
CPF (pg/g)*f*	3.17 ± 4.61 (0.09–32)
PAHs (ng/m^3^)*g*	3.37 ± 3.51 (0.50–36.5)
**a**As measured by the HOME inventory. **b**Completed years of education at child’s age 7 years. **c**As measured by TONI-3. **d**Self-reported race/ethnicity (African American = 1; Dominican = 0). **e**Self-reported ever exposed to secondhand smoke in pregnancy (yes = 1; no = 2). **f**Measured in cord blood. **g**Measured by personal air sampling.	

CPF exposure levels ranged from nondetectable to 63 pg/g. We imputed exposure levels in participants with nondetectable CPF (*n* = 115, 43%) according to assay-specific LOD values, with 93 subjects having LOD equal to 0.5 pg/g and 22 subjects having LOD equal to 1 pg/g.

*Correlation analyses for exposures and cognitive outcomes.* Unadjusted correlations between prenatal CPF exposure and log- transformed WISC-IV Composite Indices (Verbal Comprehension, Working Memory, Processing Speed, and Perceptual Reasoning), and Full-Scale IQ showed significant inverse associations between CPF exposure and *a*) Working Memory (*r* = –0.21, *p* = < 0.0001) and *b*) Full-Scale IQ (*r* = –0.13, *p* = 0.02). We observed a weak inverse correlation between CPF and Perceptual Reasoning (*r* = –0.09, *p* = 0.09), while associations of CPF with Verbal Comprehension (*r* = –0.04) and Processing Speed (*r* = –0.01) had *p*-values > 0.05.

Umbilical cord lead was not significantly correlated with CPF level (*r* = –0.08, *p* = 0.49) or WISC-IV scores (all *p*-values > 0.05) among the 89 children with lead data available. Lead was not significantly correlated with CPF level (*r* = –0.08, *p* = 0.49, as previously reported by Rauh et al. 2006) or with 7-year WISC-IV scores (all *p*-values > 0.05) among the 89 children with available data. To avoid excluding observations without lead data, we did not include lead as a covariate in regression models. ETS and (to a lesser extent) PAH were correlated with CPF (Spearman coefficients: 0.113, *p* = 0.01, and 0.07, *p* = 0.09, respectively) but were not significantly correlated (using the Mann–Whitney test for the dichotomous ETS variable) with any WISC-IV index (coefficients ranged from –0.02 to 0.03, and *p*-values ranged from 0.39 to 0.87). Birth weight was not significantly associated with any of the WISC-IV indices (all *p*-values > 0.05) and was not included in the final models.

**Figure 1:**
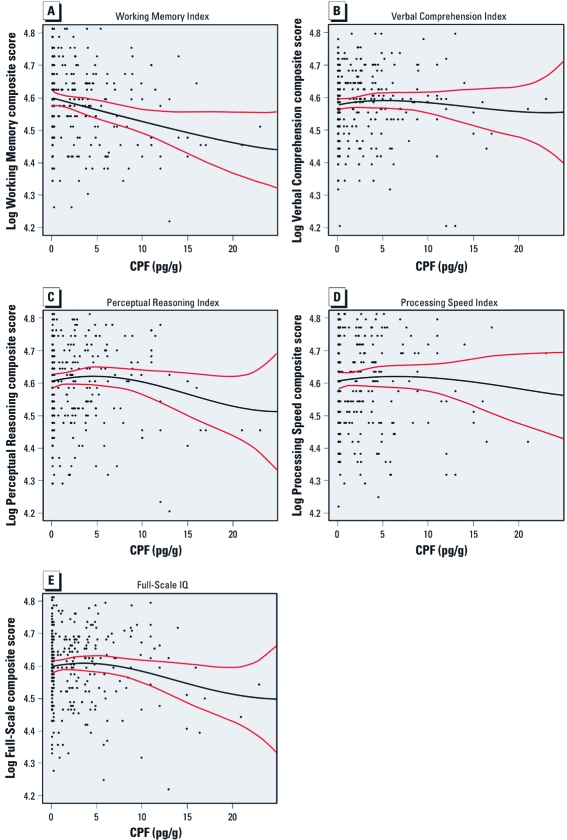
Smoothed cubic splines, superimposed over scatterplots, examining the shape of the associations between CPF exposure and (*A*) Working Memory Index, (*B*) Verbal Comprehension Index, (*C*) Perceptual Reasoning Index, (*D*) Processing Speed Index, and (*E*) Full-Scale IQ.

*Spline regression analysis.* Examination of the smoothed cubic spline regression curves, superimposed over scatterplots, indicates subtle differences in shape of the functions (Figure 1). The log-transformed Working Memory Index and Full-Scale IQ appear to be approximately linear, whereas the other functions show some curvature across exposure levels, with sparse observations at the highest exposures. Using the Davidson–MacKinnon test for comparison of non-nested models (Davidson and MacKinnon 1981), we compared models in which CPF was entered as a single continuous outcome with those in which CPF was modeled using B-splines. We failed to reject the null hypothesis that the model with CPF as a continuous measure is adequate against the alternative that the model with CPF modeled using splines provided a better fit for each WISC-IV Index (*p*-values: Verbal Comprehension Index = 0.07, Perceptual Reasoning Index = 0.08, Processing Speed Index = 0.59, Working Memory Index = 0.40, and Full-Scale IQ = 0.08).

*Estimation of linear models.* Table 2 lists the estimated B-coefficients, 95% CIs, and *p*-values for the exposure variable and covariates for the best-fitting linear regression models predicting each WISC-IV outcome. Table 2 also includes the results of linear model selection using the LASSO technique, which eliminates covariates with unstable estimates and results in more parsimonious models. Because the LASSO method uses bootstrapping to obtain standard errors, the coefficient of any covariate may be shrunk to zero if that covariate is an unstable predictor—that is, if its significance depends on the particular subset of data used in the model. The two approaches yielded very similar estimates of CPF effect. Differences in estimates for the covariates in the two methods suggest that the contribution of some covariates to WISC-IV scores may be less stable. Results for both approaches show that, on average, a 1-pg/g increase in CPF is associated with a decrease of –0.006 points in the log-transformed Working Memory score and a decrease of –0.003 points in the log-transformed Full-Scale IQ score. Because of the log transformation, estimated associations between CPF and actual Working Memory and Full-Scale IQ scores vary across the continuum of scores, such that the estimated deficit in the Working Memory score with a 1-pg/g increase in CPF ranges between 0.35 and 0.81 points, and the estimated decrease in Full-Scale IQ is between 0.20 and 0.40 points. The magnitude of these effects is more easily understood by calculating the neurodevelopmental deficit associated with an increase in CPF exposure equal to 1 SD (4.61 pg/g). On average, for each standard deviation increase in exposure, Full-Scale IQ declines by 1.4% and Working Memory declines by 2.8%. We found no significant interactions between CPF and any of the potential or final covariates, including the other chemical exposures measured during the prenatal period (ETS and PAH). Full model results for the linear regressions are provided in Supplemental Material, Table 1 (doi:10.1289/ehp.1003160), for the reader who is interested in the estimates of association between the covariates and outcomes for all of the WISC-IV index scales.

**Table 2:** Estimated associations between CPF (pg/g) and log-transformed Full-Scale IQ and each of four Composite Index scores from the WISC-IV from LASSO*a* and fully adjusted*b* linear regression models (*n* = 265).>

WISC-IV scale*^c^*	B-coefficient*^c^*	95% CI	*p*-Value
Full-Scale IQ						
LASSO		–0.003		–0.006 to 0.001		0.064
Fully adjusted		–0.003		–0.006 to 0.000		0.048
Working Memory Index						
LASSO		–0.006		–0.009 to –0.002		< 0.001
Fully adjusted		–0.006		–0.010 to –0.002		0.003
Verbal Comprehension Index						
LASSO		NA*d*		NA		NA
Fully adjusted		–0.002		–0.005 to 0.001		0.208
Perceptual Reasoning Index						
LASSO		NA		NA		NA
Fully adjusted		–0.002		–0.006 to 0.002		0.290
Processing Speed Index						
LASSO		NA		NA		NA
Fully adjusted		0.001		–0.004 to 0.005		0.728
NA, not assessed. **a**LASSO models were adjusted for maternal education, maternal IQ, and the HOME Inventory. **b**Fully adjusted models were adjusted for child sex, race/ethnicity, maternal IQ, maternal education income, child age at testing (months), ETS, and PAH. **c**All scales wereln transformed. **d**CPF was not retained in the final LASSO model.						

*Sensitivity analysis of additional influences on Working Memory Index.* To determine whether the observed CPF effect on the Working Memory Index was partially explained by its effect on general intelligence, we added the log-transformed General Ability Index, a general intelligence scale that does not include the Working Memory Index or Processing Speed Index, to the linear regression model. Although the estimate of the General Ability Index effect on Working Memory Index was significant (B-coefficient = 0.57; 95% CI,0.44–0.70; *p* < 0.001), the estimate of the CPF effect remained unchanged (–0.006), and we found no evidence of interaction between CPF and General Ability Index (*p* > 0.05), suggesting that the Working Memory effect is targeted and does not depend upon level of general intelligence.

Because child performance on the Working Memory Index can be influenced by child behavior problems (Wechsler 2003), we conducted a supplementary analysis to rule out the possibility that the observed associations between CPF and the Working Memory Index might be affected by behavior problems, as measured by the clinically oriented diagnostic and statistical manual scales on the Child Behavior Checklist. We found no evidence of indirect “mediation” using Sobel’s test, with *p*-values ranging from 0.31 to 0.99 (MacKinnon et al. 2002; Sobel 1982). Full model results are provided in Supplemental Material, Table 2 (doi:10.1289/ehp.1003160), for the reader who is interested in the estimates of association between child behavior problems and Working Memory Index.

*Sensitivity analysis of the influence of LOD imputation.* After obtaining all results, we recomputed all estimates of association between CPF and WISC-IV scores among subjects with detectable CPF levels only. Analysis with detects alone is known to give unbiased estimates of the parameters of interest (Little 1992). In the present sample, we observed no consistent differences in estimates when we excluded imputed CPF data (data not shown).

## Discussion

Results of this study showed that higher prenatal CPF exposure, as measured in umbilical cord blood plasma, was associated with decreases in cognitive functioning on two different WISC-IV indices, in a sample of urban minority children at 7 years of age. Specifically, for each SD increase in exposure (4.61 pg/g), Full-Scale IQ declined, on average, by 1.4% (0.94–1.8 points) and Working Memory Index scores declined by 2.8% (1.6–3.7 points). The dose–effect relationships between CPF exposure and log-transformed Working Memory Index and Full-Scale IQ scores are linear across the range of exposures in the study population, with no evidence for a threshold. Of the WISC-IV indices used as end points, the Working Memory Index was the most strongly associated with CPF exposure in this population.

Although no other epidemiologic studies have evaluated the neurotoxicity of prenatal CPF exposure on cognitive development at the time of school entry, several prior studies, using the present biomarker of exposure, have reported evidence of early cognitive and behavioral effects associated with a urinary biomarker of nonspecific OP exposure (Engel et al. 2007; Eskenazi et al. 2007; Young et al. 2005). Outcomes associated with exposure in these studies, as well as in our own earlier work (Rauh et al. 2006), have included attentional problems (e.g., Marks et al. 2010). These prior findings are consistent with the present 7-year results, because working memory skills involve attentional processes. More important, problems in working memory may interfere with reading comprehension, learning, and academic achievement, although general intelligence remains in the normal range (Blair 2006). Working memory is less likely than full-scale IQ to be affected by socioeconomic or cultural conditions (Wechsler 2003), providing a useful, more targeted measure of possible neurotoxic effects on brain function.

Several different theories or models address how working memory operates in the human brain, but most agree that it involves a system of limited attention capacity, supplemented by more peripherally based storage systems (Baddeley and Logie 1999). Some theories emphasize the role of attentional control in working memory (e.g., Cowan 1999), whereas others stress a multicomponent model, including a control system of limited attentional capacity (the central executive control system), assisted by phonological and visuospatial storage systems (see review by Baddeley 2003). To date, most studies of the anatomical localization of working memory problems are based on clinical populations (individuals with specific brain lesions) (Vallar and Pagano 2002) and some neuroimaging studies in small numbers of normal subjects (Smith and Jonides 1997). More refined neuropsychological tests and neuroimaging studies are needed to determine whether CPF-related working memory deficits are primarily auditory (part of a phonological loop with implications for language acquisition) or primarily related to visuospatial short-term memory (reflecting nonverbal intelligence tasks).

Few human studies have focused on possible mechanisms underlying neurodevelopmental deficits associated with OP exposure, but there is evidence that certain genetic polymorphisms can affect CPF metabolism (e.g., Berkowitz et al. 2004). Such findings suggest that some populations may be more vulnerable and may exhibit adverse neurodevelopmental effects at much lower exposures than other populations (Berkowitz et al. 2004). Again, neuroimaging studies would be useful to determine if population differences in vulnerability to CPF are also reflected in population differences in brain abnormalities associated with exposure.

Although behavioral alterations observed in rodents may be imperfect analogues for humans, they have guided human studies by identifying specific deficits in locomotor activity, learning, and memory (e.g., Aldridge et al. 2005). In light of experimental evidence suggesting that CPF effects in rodents may be irreversible (Slotkin 2005), it will be important to determine how any neurocognitive deficits associated with prenatal CPF exposure might respond to treatment or early intervention. Here, we may benefit from studies of lead-exposed children that have demonstrated evidence of reversals in learning deficits as a result of environmental enrichment (Guilarte et al. 2003).

Some limitations of this study should be noted. Our sample consists of low-income, urban, minority children who may experience other unmeasured exposures or underlying health problems that could potentially confound or modify associations with pesticide exposure. Furthermore, in the absence of firm mechanistic evidence linking brain anomalies to more refined neuropsychological testing, the observed functional deficits at 7 years of age should be interpreted with caution. We cannot directly compare our findings with the results from the other epidemiological studies that have relied on urinary OP concentrations as the biomarker of exposure.

In June 2000, the U.S. EPA announced a phase-out of the sale of CPF for indoor residential use, with a complete ban effective 31 December 2001 (U.S. EPA 2000, 2002). After the ban, levels of CPF in personal and indoor air samples in our own cohort decreased by more than 65%, and plasma blood levels dropped by more than 80% (Whyatt et al. 2005), despite some lingering residential residues (Whyatt et al. 2007). From other parts of the country, there is evidence of continued low-dose exposures in children from food residues (Lu et al. 2006). Because agricultural use of CPF is still permitted in the United States, it is important that we continue to monitor the levels of exposure in potentially vulnerable populations, including pregnant women in agricultural communities, and evaluate the long-term neurodevelopmental implications of exposure to CPF and other OP insecticides.

## Supplemental Material

(112 KB) PDF
