# Supplementary material for: Seven-Year Neurodevelopmental Scores and Prenatal Exposure to Chlorpyrifos, a Common Agricultural Pesticide
Source: Environ Health Perspect. 2011 Apr 21;119(8):1196–201. doi: 10.1289/ehp.1003160 (PMC3237355; doi:10.1289/ehp.1003160)
Supplement: (112 KB) PDF [file ehp.1003160.s001.pdf]

Supplemental Material

Title: 7-Year Neurodevelopmental Consequences of Prenatal Exposure to Chlorpyrifos, a Common Organophosphate Pesticide

Authors:

Virginia Rauh
Srikesh Arunajadai
Megan Horton
Frederica Perera
Lori Hoepner
Dana B. Barr
Robin Whyatt

Supplemental Material Table 1. Linear regression models with estimated coefficients and 95% CIs in points on log-transformed WISC-IV Indexes for each unit increase in chlorpyrifos (CPF) exposure level (pg/g), adjusted for all covariates, at child age 7 years

	Full-Scale IQ			Working Memory			Verbal Comprehension			Perceptual Reasoning			Processing Speed		
Covariate	B	95% CI	p-Value	B	95% CI	p-Value	B	95% CI	p-Value	B	95% CI	p-Value	B	95% CI	p-Value
CPF	-0.003	-0.006, 0.000	0.05	-0.006	-0.010, -0.002	<0.01	-0.002	-0.005, 0.001	0.21	-0.002	-0.006, 0.002	0.29	0.001	-0.004, 0.005	0.73
HOME	0.001	-0.002, 0.004	0.46	0.000	-0.003, 0.004	0.810	0.003	0.000, 0.005	0.03	0.001	-0.002, 0.004	0.60	-0.002	-0.006, 0.002	0.24
Sex	-0.038	-0.067, -0.010	0.01	-0.040	-0.075, -0.005	0.03	-0.018	-0.045, 0.009	0.18	-0.011	-0.044, -0.022	0.50	-0.063	-0.103, -0.024	<0.01
Maternal Education	0.006	0.000, 0.012	0.04	0.002	-0.005, 0.010	0.50	0.005	0.000, 0.011	0.07	0.007	0.000, 0.014	0.04	0.006	-0.003, 0.014	0.18
Maternal IQ	0.001	0.000, 0.002	0.02	0.001	-0.001, 0.002	0.38	0.001	0.000, 0.002	0.08	0.002	0.001, 0.003	<0.01	0.000	-0.002, 0.002	0.97
Race/ ethnicity	0.006	-0.024, 0.036	0.71	0.003	-0.034, 0.040	0.87	0.040	0.011, 0.068	<0.01	- 0.017	-0.052, 0.018	0.33	-0.014	-0.055, 0.028	0.52
Income	0.030	0.008, 0.051	0.01	0.026	-0.001, 0.053	0.05	0.022	0.001, 0.043	0.04	0.022	-0.003, 0.048	0.08	0.014	-0.016, 0.044	0.35
ETS	-0.004	-0.034, 0.026	0.80	-0.007	-0.044, 0.030	0.70	0.019	-0.010, 0.047	0.20	-0.013	-0.048, 0.022	0.45	-0.016	-0.058, 0.026	0.45
PAH	0.001	-0.020, 0.022	0.92	-0.002	-0.028, 0.023	0.85	0.004	-0.016, 0.023	0.72	0.002	-0.022, 0.026	0.84	-0.010	-0.039, 0.019	0.48
Child age at testing	-0.001	-0.006, 0.005	0.74	0.000	-0.006, 0.007	0.95	-0.004	-0.009, 0.001	0.14	0.001	-0.005, 0.007	0.76	0.000	-0.07, 0.008	0.96

[a] Home Observation for Measurement of the Environment
[b] Sex of child: female =1; male =0
[c] Completed years of maternal education at child age 7 years
[d] Test of Non-Verbal Intelligence-Third Edition (TONI-3)
[e] Race/Ethnicity: African American=1, Dominican=0
[f] Less than $20,000 annual income
[g] Self-reported ever exposed to secondhand smoke in pregnancy (yes=1; no=2), validated by cord blood cotinine level
[h] Polycyclic aromatic hydrocarbons measured by personal air sampling
[i] Exact child age at testing in months

Supplemental Material Table 2. Sobel's Test of Mediation by child behaviors[a] on the association between prenatal CPF exposure and log Working Memory Index

Covariate (n=265)	Separate models estimating CPF effect on Working Memory Index in the presence of each covariate			Separate models estimating the effect of each covariate on Working Memory Index in the presence of CPF			Sobels's Test of Mediation
	Estimate	95% CIs	p-Value	Estimate	95% CIs	p-Value	
Behavior							
Anxiety	-0.006	-0.01, -0.002	0.001	-0.047	-0.071,-0.023	<0.001	0.53
Affective	-0.007	-0.01, -0.003	<0.001	-0.044	-0.072, -0.016	0.001	0.34
ADHD	-0.006	-0.01, -0.002	0.001	-0.018	-0.026, -0.010	<0.001	0.72
Conduct	-0.006	-0.01, -0.002	0.002	-0.047	-0.071, -0.023	<0.001	0.31
Oppositional	-0.006	-0.01, -0.002	0.001	-0.023	-0.041, -0.005	0.012	0.84
Somatic	-0.006	-0.01, -0.002	0.001	-0.001	-0.050, 0.050	0.986	0.99

[a]All behaviors are from the Diagnostic and Statistical Manual problem scales on the Child Behavior Checklist (6-18 years), parent version, at child age 7 years.
